# Evaluating the intersection of climate vulnerability and cancer burden in North Carolina

**DOI:** 10.1093/jncics/pkae124

**Published:** 2024-12-18

**Authors:** Joyce Pak, Ngan Le, Eman M Metwally, Jeanny Wang, Arrianna Marie Planey, Amy M Lowman, Bradford E Jackson, Eboneé N Butler, Jennifer L Lund

**Affiliations:** Department of Epidemiology, University of North Carolina at Chapel Hill, Chapel Hill, NC 27516, United States; Department of Epidemiology, University of North Carolina at Chapel Hill, Chapel Hill, NC 27516, United States; Department of Epidemiology, University of North Carolina at Chapel Hill, Chapel Hill, NC 27516, United States; Department of Epidemiology, University of North Carolina at Chapel Hill, Chapel Hill, NC 27516, United States; Department of Health Policy and Management, University of North Carolina at Chapel Hill, Chapel Hill, NC 27516, United States; Lineberger Comprehensive Cancer Care Center, University of North Carolina at Chapel Hill, Chapel Hill, NC 27516, United States; Carolina Population Center, University of North Carolina at Chapel Hill, Chapel Hill, NC 27516, United States; Department of Epidemiology, University of North Carolina at Chapel Hill, Chapel Hill, NC 27516, United States; Lineberger Comprehensive Cancer Care Center, University of North Carolina at Chapel Hill, Chapel Hill, NC 27516, United States; Department of Epidemiology, University of North Carolina at Chapel Hill, Chapel Hill, NC 27516, United States; Lineberger Comprehensive Cancer Care Center, University of North Carolina at Chapel Hill, Chapel Hill, NC 27516, United States; Department of Epidemiology, University of North Carolina at Chapel Hill, Chapel Hill, NC 27516, United States; Lineberger Comprehensive Cancer Care Center, University of North Carolina at Chapel Hill, Chapel Hill, NC 27516, United States

## Abstract

Climate-related extreme weather events disrupt health-care systems and exacerbate health disparities, particularly affecting individuals diagnosed with cancer. This study explores the intersection of climate vulnerability and cancer burden in North Carolina (NC). Using county-level data from the US Climate Vulnerability Index (CVI) and the NC Department of Health and Human Services, we analyzed cancer incidence and mortality rates from 2017 to 2021. Our findings reveal a robust correlation between CVI percentiles and cancer mortality (*r* = 0.72). Counties with high area deprivation like Scotland, Robeson, and Halifax had the highest CVI percentiles of 0.68, 0.67, and 0.66, with respective cancer mortality rates of 193, 195, and 196 per 100 000 person-years. Correlations between CVI and cancer incidence were modest (*r* = 0.22). These results underscore the need for targeted public health interventions to mitigate climate-related health disparities. Future work could focus on exploring specific climate hazards and cancer outcomes to enhance preparedness and resilience in cancer care.

## Background 

Climate-related extreme weather events lead to major disruptions in health-care systems and populations that depend on these systems for managing chronic disease.^1^ Individuals diagnosed with cancer are among the most vulnerable populations in the aftermath of disaster, as extreme weather events can disable transportation, communication, and power systems.^1^ This may impede patients’ access to cancer care and hinder cancer treatment facilities’ ability to deliver care.^1-3^ Consequently, this can result in delays in early detection, diagnosis, and treatment, leading to adverse outcomes such as late-stage diagnosis, decreased treatment adherence, and increased mortality rates.^1-3^ Furthermore, medical surge capacities are limited, resulting in overtaxed medical services and spillover effects on surge capacity at facilities serving populations who have left impact areas for safe zones.^4^

The communities most vulnerable to extreme weather events are often the same communities with the highest levels of poverty, an issue of increasing national and international importance.^2^^,^^5^ In places like Eastern North Carolina (NC), home to some of the most racially diverse and poorest rural communities in the United States, including large Black, Hispanic, and state-recognized Indian tribes,^6^ cancer death rates exceed the national average by 16%.^7^ These extreme weather events exacerbate existing health and health-care disparities.^7^

The relationship between climate-related impacts and cancer burden in NC has not been explored. We used publicly available, aggregated, county-level measures of climate and cancer data to evaluate intersections between climate vulnerability and cancer burden by describing geographic variation in county-level cancer incidence and mortality.

## Methods

### Data source and population data

We conducted an analysis using county-level baseline vulnerabilities and climate change risk data from the US Climate Vulnerability Index (CVI), alongside cancer incidence and mortality period data from 2017 to 2021, as reported by the NC Department of Health and Human Services.^8^ We used the National Center for Health Statistics 2013 Urban-Rural Classification Scheme and collapsed the county-level population into large urban (large central metropolitan, large fringe metropolitan), medium urban (medium metropolitan, small metropolitan), and rural (micropolitan, noncore) counties. Furthermore, we calculated Area Deprivation Index (ADI) rank scores as a measure of socioeconomic disadvantage, accounting for income, education, employment, and availability of quality housing.^9^

We used the CVI dataset from the Environmental Defense Fund and Texas A&M University, a data-driven mapping tool that provides a comprehensive and quantitative assessment of 4 baseline vulnerabilities (health, social/economic, infrastructure, and environment) and 3 climate change-related disparities (health, social/economic, extreme events) across the United States.^10^ By integrating data at the census tract level, the CVI systematically ranks more than 70 000 US Census tracts according to their vulnerability to climate change effects, using 184 indicators to characterize the geographic distribution of health, social, environmental, and climate disparities across the country; data are subsequently aggregated to the county level.^10^ For each county, a percentile rank ranging from 0.00 to 1.00 was generated, with higher values indicating greater climate vulnerability. Our study aimed to describe the impact of the CVI on age-adjusted cancer incidence and mortality rates across different counties.

### Statistical analyses

We estimated county-level age-adjusted cancer incidence rates (AAIR) and mortality rates (AAMR) per 100 000 person-years with 95% confidence intervals (CIs) for the overall population and stratified by cancer type and rurality. Direct age adjustments were applied to crude incidence and mortality rates using the 2000 US population.^8^

Continuous variables (eg, counties’ attributes used in the CVI) are reported as median (interquartile range [IQR]). We classified the percentile rankings for CVI into quartiles: first (most favorable, 0.00-0.25) to fourth (least favorable, 0.75-1.00). We aggregated counties across CVI quartiles to compare incidence and mortality rates among quartiles. To obtain aggregated data, we included all counties regardless of population size and death counts.

Rate ratios (RRs) were estimated by comparing county-specific incidence and mortality rates between the fourth and first CVI quartiles for the overall population and stratified by cancer type and rurality. Univariate Poisson regression was used to calculate RRs and corresponding 95% CIs. All analyses were conducted using R version 4.3.2, and the study was approved by the University of North Carolina’s (UNC) Institutional Review Board (IRB #: 23-1749).

## Results

Across the 100 counties in NC, the age-adjusted cancer incidence ranged from 261 to 535 (median = 471, IQR = 442-493) and mortality ranged from 125 to 229 (median = 162, IQR = 151-173) per 100 000 person-years. All-cancer incidence was modestly correlated with CVI (*r* = 0.22), whereas breast cancer incidence was negatively correlated with CVI (*r* = −0.11). Notably, there was a strong correlation between CVI and ADI (*r* = 0.90). A robust correlation was identified between CVI and cancer mortality (*r* = 0.72), revealing communities more vulnerable to climate threats bear a disproportionate burden of cancer mortality. Scotland, Robeson, and Halifax counties, areas with high area-level deprivation,^11^ had the highest overall CVI scores of 0.68, 0.67, and 0.66, and cancer mortality rates of 193, 195, and 196 per 100 000 person-years, respectively. These counties are located in Eastern NC, a region characterized by well-documented health disparities, including poorer health outcomes, lower quality of life, and socioeconomic challenges such as higher unemployment and elevated poverty rates, as highlighted by the County Health Rankings.^11^ Subgroup analysis by cancer site revealed the strongest correlations between CVI and cancer mortality from colorectal (*r* = 0.61), lung (*r* = 0.56), and breast (*r* = 0.39), respectively.

**Figure 1.**
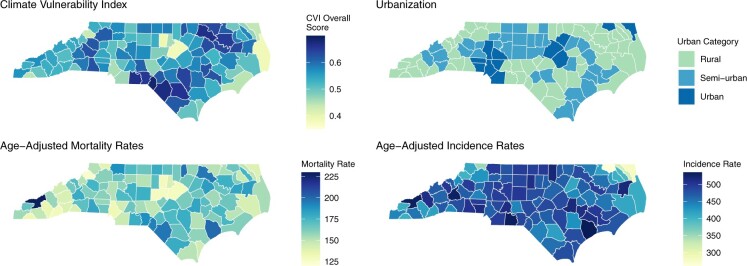
Geographical distribution of climate vulnerability, urbanization, and age-adjusted mortality and incidence rates across North Carolina. CVI = Climate Vulnerability Index.

Between 2017 and 2021, the AAMR for cancer was 162.4 (95% CI = 158.8 to 166) per 100 000 person-years. The AAMR was the highest in the fourth quartile (174.3; 95% CI = 168.4 to 180.2) and lowest in the first quartile (145.6; 95% CI = 140.6 to 150.5), accounting for 29 excess deaths per 100 000 person-years in the fourth vs first quartile (RR = 1.2; 95% CI = 1.15 to 1.25). Figure 1 illustrates the distribution of AAMR and AAIR for cancer, CVI, and urbanization across NC. The AAMR increased across rurality from the lowest to highest CVI quartiles (Table 1). Across all categories of cancer site and rurality, AAIR showed a relatively small increase when comparing the first and fourth CVI quartiles (overall RR = 1.07; 95% CI = 1.05 to 1.1) (Table 1).

**Table 1.** Age-adjusted mortality and incidence rates for cancer stratified by CVI quartiles.

		Total	Q1	Q2	Q3	Q4	Rate ratio; Q4/Q1
**Mortality**							
	All cancer	162.4 (158.8 to 166.0)	145.6 (140.6 to 150.5)	157.5 (151.3 to 163.8)	172.3 (166.5 to 178.1)	174.29 (168.4 to 180.2)	1.20 (1.15 to 1.25)
*Cancer type*							
	Lung	41.8 (40.3 to 43.3)	35.1 (32.3 to 37.8)	41.4 (38.6 to 44.2)	46.0 (43.8 to 48.2)	44.8 (42.2 to 47.3)	1.28 (1.17 to 1.40)
	Female breast	21.3 (20.5 to 22.1)	19.2 (18.1 to 20.3)	21.2 (19.4 to 23.0)	21.1 (19.5 to 22.7)	23.3 (21.5 to 25.1)	1.21 (1.06 to 1.39)
	Prostate	20.6 (19.7 to 21.5)	19.2 (18.1 to 20.3)	19.5 (18.0 to 20.9)	20.9 (18.6 to 23.3)	22.4 (20.3 to 24.4)	1.17 (1.01 to 1.35)
	Colorectal	14.1 (13.4 to 14.8)	11.7 (10.9 to 12.4)	13.3 (12.3 to 14.2)	14.9 (13.3 to 16.4)	16.1 (14.5 to 17.8)	1.38 (1.17 to 1.64)
*Urbanization*							
	Large metropolitan	152.7 (143.5 to 161.9)	144.2 (132.6 to 155.8)	161.3 (151.2 to 171.4)	169.5 (126.9 to 212.0)	—	—
	Medium small metro	161.2 (155.7 to 166.7)	147.0 (134.5 to 159.4)	159.2 (148.8 to 169.6)	171.3 (166.1 to 176.5)	170.6 (158.2 to 182.9)	1.16 (1.06 to 1.27)
	Micropolitan/noncore	165.3 (160.0 to 170.7)	145.4 (137.9 to 152.8)	154.3 (142.7 to 165.9)	173.3 (162.7 to 183.9)	175.2 (168.0 to 182.4)	1.21 (1.13 to 1.28)
**Incidence**							
	All cancer	462.5 (453.2 to 471.7)	439.2 (412.8 to 465.7)	463.7 (446.2 to 481.2)	475.7 (461.2 to 490.3)	471.1 (458.4 to 483.8)	1.07 (1.05 to 1.10)
*Cancer type*							
	Lung	63.3 (61.2 to 65.4)	54.0 (49.8 to 58.1)	65.0 (61.0 to 69.0)	67.4 (63.7 to 71.1)	66.7 (63.2 to 70.2)	1.24 (1.15 to 1.33)
	Female breast	154.4 (149.8 to 159.1)	151.7 (138.9 to 164.4)	158.1 (149.0 to 167.2)	153.6 (146.5 to 160.7)	154.3 (145.3 to 163.3)	1.02 (0.97 to 1.06)
	Prostate	114.9 (111.3 to 118.5)	110.9 (102.7 to 119.2)	111.5 (105.5 to 117.6)	113.1 (115.6 to 132.9)	124.2 (115.6 to 132.9)	1.12 (1.06 to 1.18)
	Colorectal	35.3 (34.0 to 36.5)	31.1 (29.1 to 33.2)	33.3 (30.9 to 35.7)	37.3 (34.7 to 39.9)	39.4 (37.5 to 41.3)	1.27 (1.15 to 1.39)
*Urbanization*							
	Large metropolitan	448.2 (395.3 to 501.1)	418.2 (327.6 to 508.8)	481.9 (455.2 to 508.7)	502.6 (461.9 to 543.2)	—	—
	Medium small metro	480.0 (471.9 to 488.1)	478.0 (471.5 to 484.5)	480.1 (462.6 to 497.6)	480.5 (457.8 to 503.3)	482.0 (460.2 to 503.8)	1.01 (0.96 to 1.06)
	Micropolitan/noncore	454.6 (442.5 to 466.6)	423.0 (390.3 to 455.7)	438.5 (402.2 to 474.8)	468.8 (446.2 to 491.5)	468.4 (453.0 to 483.7)	1.11 (1.07 to 1.15)

There were no large metropolitan counties in the fourth quartile for CVI; therefore, no estimates are reported. Age-adjusted mortality and incidence rates are presented per 100 000 persons with 95% confidence interval. CVI = Climate Vulnerability Index

## Discussion

Our study demonstrates the graded increase in county-level cancer mortality with greater climate vulnerability. Furthermore, the incremental impact of climate vulnerability was higher for mortality and incidence when stratified by cancer type and rurality. This finding supports previous reports of climate vulnerability in rural communities across the United States. For example, Hurricane Maria’s (2017) landfall in Puerto Rico resulted in a disproportionate number of long-duration power failures in rural communities, accounting for 61% of all power loss.^12^ Similarly, when Hurricane Beryl swept across Texas in July 2024, widespread power outages led to local hospitals reporting a spike in heat-related illnesses, with at least 7 people dying from extreme heat.^13^ Furthermore, much of Hurricane Helene’s (2024) damage affected NC areas with high percentage of older residents and rural communities with limited access to health-care and social services.^14^ Many residents in this region rely on government-subsidized programs for food, medical care, and housing, with more than 12% living in poverty.^14^ Higher risk populations will continue to be negatively affected by climate change unless disparities are addressed through targeted mitigation strategies, especially low-wealth and climate-vulnerable communities.^15^

To the best of our knowledge, no prior studies have assessed the association between CVI and cancer burden in NC. While there was a strong correlation between CVI and cancer mortality, there was only a modest correlation with CVI and all-cancer incidence and an inverse correlation with CVI and breast cancer incidence. This builds on prior findings of a strong inverse association between ADI and breast cancer screening.^16^ Individuals from census block groups in the lowest ADI quintile were about half as likely to undergo recommended cancer screening as those living in the highest.^16^ Implementing area-based measures (eg, ADI) may help inform interventions seeking to address disparities based on social determinants of health and climate vulnerability.^16^^,^^17^

The strong association between climate vulnerability and cancer mortality underscores previous reports of the deleterious effects of climate change on the cancer care continuum. Several studies^18-21^ reported interruption in diagnosis and treatment of cancer during natural disasters, which negatively affect patient survival.^22^ While patients with cancer are uniquely vulnerable to care disruption, they are often deprioritized during scarce health-care disaster response allocation.^23^ The problem is compounded among historically marginalized communities, who already suffer from entrenched racial and socioeconomic cancer disparities. Therefore, cancer-specific emergency preparedness plans (ie, ensuring refrigerated storage for chemotherapy drugs, prioritizing backup generators for treatment centers, and training health-care staff in disaster response)^1^^,^^24^ are crucial to mitigate interruptions in cancer care delivery and increases in cancer care disparities.^25^^,^^26^ The CVI’s comprehensive approach to integrating area-level health, socioeconomic, and environmental factors can be a novel metric for community-level measures of vulnerability to climate change.^10^

### Limitations

Although the CVI is a comprehensive measure, it is by no means inclusive of all climate change risks and inequalities of health, social, infrastructure, and environmental quality.^10^ Second, the CVI relies heavily on self-reported, survey-based data for any health-based metrics developed by the Centers for Disease Control and Prevention (CDC), making it susceptible to recall and nonresponse biases.^10^ Furthermore, the CVI is based on a snapshot in time, which might not be optimal to inform policy makers.^10^ Having annual CVI data would provide more dynamic insights to understand changes in health impacts of climate change over time. Additionally, our analysis shows that the CVI and measures of social vulnerability (eg, ADI) are strongly correlated. However, the CVI remains unique in its inclusion of climate change risk domains (eg, the health, social, and economic costs of extreme events) that capture additional dimensions of vulnerability. Last, we conducted a cross-sectional analysis and therefore could not infer causal relationships between county-level characteristics and mortality. Future work could explore associations between specific climate hazards and cancer outcomes to enhance equitable climate preparation, mitigation, and adaptation strategies. By mapping cancer mortality rates to the climate hazards relevant to NC, cancer centers and hospitals can better prepare for extreme weather events and direct public health resources to the most affected people and places.

## Conclusion

This study demonstrates a graded increase in cancer mortality in NC counties with worsening climate vulnerability. These findings emphasize the need for improved resource allocation strategies and targeted climate-related public health interventions to address climate injustice and improve cancer outcomes, with implications extending beyond NC to vulnerable communities across the United States and globally.

## Data Availability

The datasets generated and analyzed during this study are publicly available. The data can be accessed at the Climate Vulnerability Index repository at https://climatevulnerabilityindex.org/. The second dataset is available from the North Carolina State Center for Health Statistics at https://schs.dph.ncdhhs.gov/data/cancer.cfm. No special permissions are required to access these data.
